# Paracrine interactions between mesenchymal stem cells and macrophages are regulated by 1,25-dihydroxyvitamin D3

**DOI:** 10.1038/s41598-017-15217-8

**Published:** 2017-11-03

**Authors:** Laura Saldaña, Gema Vallés, Fátima Bensiamar, Francisco José Mancebo, Eduardo García-Rey, Nuria Vilaboa

**Affiliations:** 1 Hospital Universitario La Paz-IdiPAZ, Paseo de la Castellana 261, 28046 Madrid, Spain; 2 CIBER de Bioingeniería, Biomateriales y Nanomedicina (CIBER-BBN), Madrid, Spain

**Keywords:** Tissue engineering, Mesenchymal stem cells, Mesenchymal stem cells, Tissue engineering

## Abstract

Mesenchymal stem cells (MSC) modulate the macrophage-mediated inflammatory response through the secretion of soluble factors. In addition to its classical effects on calcium homeostasis, 1,25-dihydroxyvitamin D3 (1,25D3) has emerged as an important regulator of the immune system. The present study investigates whether 1,25D3 modulates the paracrine interactions between MSC and macrophages. 1,25D3 stimulated MSC to produce PGE_2_ and VEGF and regulated the interplay between macrophages and MSC toward reduced pro-inflammatory cytokine production. Conditioned media (CM) from co-cultures of macrophages and MSC impaired MSC osteogenesis. However, MSC cultured in CM from 1,25D3-treated co-cultures showed increased matrix maturation and mineralization. Co-culturing MSC with macrophages prevented the 1,25D3-induced increase in RANKL levels, which correlated with up-regulation of OPG secretion. MSC seeding in three-dimensional (3D) substrates potentiated their immunomodulatory effects on macrophages. Exposure of 3D co-cultures to 1,25D3 further reduced the levels of soluble factors related to inflammation and chemotaxis. As a consequence of 1,25D3 treatment, the recruitment of monocytes toward CM of 3D co-cultures decreased, while the osteogenic maturation of MSC increased. These data add new insights into the pleiotropic effects of 1,25D3 on the crosstalk between MSC and macrophages and highlight the role of the hormone in bone regeneration.

## Introduction

Vitamin D plays an important role in maintaining bone integrity, as its deficiency has been related to impaired bone fracture healing and implant osseointegration^1,2^. Many biological actions of calcitriol (1,25-dihydroxyvitamin D3, 1,25D3) are mediated by the vitamin D receptor (VDR). Upon ligand binding, VDR heterodimerizates with the nuclear retinoid X receptor (RXR), and the resulting VDR-RXR complex can bind to specific DNA sequences, termed vitamin D-response elements (VDRE), located in the promoters of target genes^3^. Although VDR is expressed in bone tissue, direct effects of 1,25D3 on osteoblast-lineage cells are not yet fully defined. The actions of 1,25D3 on immature osteoblasts and osteoprogenitors are mainly directed toward maintaining serum calcium levels by increasing bone resorption through the regulation of receptor activator of NF-κB ligand (RANKL)^4^. On the other hand, 1,25D3 stimulates *in vitro* osteoblast differentiation and mineralization as well as mineral deposition in osteoid tissues, as evidenced in transgenic mice with osteoblast-specific overexpression of VDR^5,6^.

Beyond its well-documented role in regulating calcium and bone homeostasis, 1,25D3 has attracted considerable attention due to its immunoregulatory functions. Vitamin D deficiency has been associated with increased autoimmunity and susceptibility to infection^7^. Cells of the immune system, including promyelocytes, monocytes and dendritic cells express VDR and are capable of responding to 1,25D3^8^. VDR-mediated signaling in immune cells results in the activation of downstream gene expression, which ultimately elicits anti-proliferative and immunomodulatory effects^9^. In cells of the monocyte/macrophage lineage, 1,25D3 decreases the production of inflammatory cytokines, chemokines and prostaglandins^10,11^. Macrophages are key mediators of osseous wound healing. They participate in the initial inflammatory phase following tissue injury and are essential for bone healing via secretion of inflammatory, osteotropic and pro-angiogenic factors. In bone tissue engineering procedures, macrophages facilitate new bone formation around the implanted scaffolds, although excessive macrophage activation has been associated with the development of a fibrous capsule that confines the implant and impairs its physical connectivity with the host bone^12^.

Tissue engineering combines scaffolds and mesenchymal stem cells (MSC) to enhance regeneration of injured tissues. MSC modulate both the inflammatory reaction and the subsequent reparative phases of wound healing, mainly through the secretion of soluble factors^13^. Based on *in vitro* and *in vivo* studies of inflammatory disorders, it has become clear that MSC modulate macrophage functions. Co-culturing with MSC drives macrophages toward a regulatory phenotype via increased expression of interleukin-10 (IL-10), IL-4 and reduced production of pro-inflammatory cytokines such as tumor necrosis factor-α (TNF-α), IL-6, IL-12, IL-1β and monocyte chemoattractant protein-1 (MCP-1)^14^. MSC cultured on three-dimensional (3D) polymeric substrates or encapsulated in synthetic hydrogels lessen *in vitro* macrophage activation^15,16^. Moreover, MSC encapsulated within hydrogels reduce the thickness of the fibrous capsule upon subcutaneous implantation in mice, compared with acellular hydrogels^15^. Several lines of evidence suggest that 1,25D3 might regulate cellular events during bone regeneration. After intravenous administration, 1,25D3 accumulates in the callus during rat femoral fracture healing and a vitamin D-rich diet improves peri-implant bone formation in ovariectomized rats^17,18^. The present study investigated whether 1,25D3 modulates the paracrine interactions between MSC and macrophages. To this end, co-cultures of macrophages and MSC seeded on flat surfaces or 3D scaffolds were exposed to 1,25D3, and levels of soluble factors related to inflammation and bone remodeling were quantified. A comparative evaluation of 1,25D3 effects on single-cultured and co-cultured cells was performed. The influence of soluble factors contained in conditioned media (CM) from co-cultures treated with 1,25D3 on monocyte migration and functionality of MSC and osteoblasts was also evaluated.

## Results

### Cell responsiveness to 1,25D3

**Figure 1:**
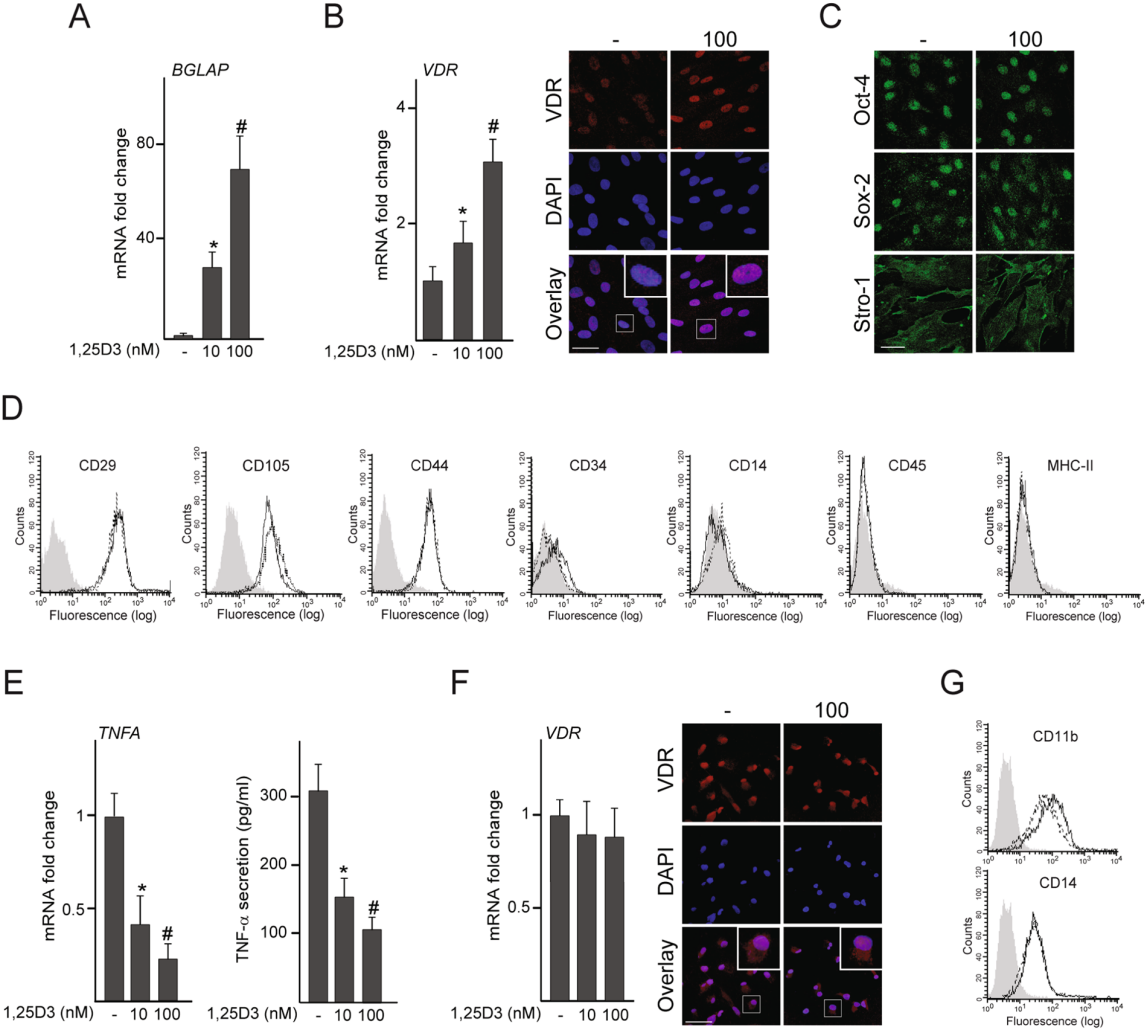
Cell response to 1,25D3. MSC and dTHP-1 were treated with 10 or 100 nM 1,25D3 or vehicle (−) for 72 h. *BGLAP* mRNA fold changes in MSC (**A**). *VDR* mRNA fold changes in MSC (**B**) or dTHP-1 (**F**) (left panels) and confocal images showing double staining of VDR (red), nuclei (blue) and overlay (right panels). Boxed areas are magnified in the upper right corner. Confocal images showing MSC stained for Oct-4, Sox-2 and Stro-1 (green) (**C**). Flow cytometric determinations of the expression of surface markers in MSC (**D**) or dTHP-1 (**G**) treated with 100 nM 1,25D3 (solid lines) or vehicle (dotted lines). Gray filled histograms correspond to negative controls. *TNFA* mRNA fold changes (left panel) and TNF-α secretion (right panel) in dTHP-1 (**E**). mRNA data are relative to those measured in untreated cells, which were given the arbitrary value of 1. *p < 0.05 compared with untreated cells; ^#^p < 0.05 compared with 10 nM 1,25D3. Bar = 100 μm.

In a first set of experiments, we studied the responsiveness of MSC and THP-1 cells treated with 12-O tetradecanoyl phorbol 13-acetate (TPA) (dTHP-1) to 1,25D3 by quantification of the transcript levels of the genes encoding for the bone gamma-carboxyglutamate protein (BGLAP) and TNF-α, which contain VDRE in their promoters^19,20^. Treatment of MSC with 1,25D3 for 72 h highly increased *BGLAP* mRNA levels in a dose-dependent manner (Fig. 1A). *VDR* mRNA levels and VDR nuclear staining also increased after 1,25D3 exposure (Fig. 1B). Treatment with 100 nM 1,25D3 did not affect MSC identity, as assessed by examination of stemness markers and expression profile of specific cell-surface molecules (Fig. 1C,D). Exposure of dTHP-1 to 1,25D3 reduced TNF-α mRNA levels and secretion in a dose-dependent manner (Fig. 1E). In this cell type, *VDR* mRNA levels and VDR nuclear staining were not affected by the hormone (Fig. 1F). Treatment with 1,25D3 also did not affect the expression levels of surface markers related to the monocyte/macrophage lineage (Fig. 1G).

### Effect of 1,25D3 on levels of inflammation-related factors in MSC/dTHP-1 co-cultures

**Figure 2:**
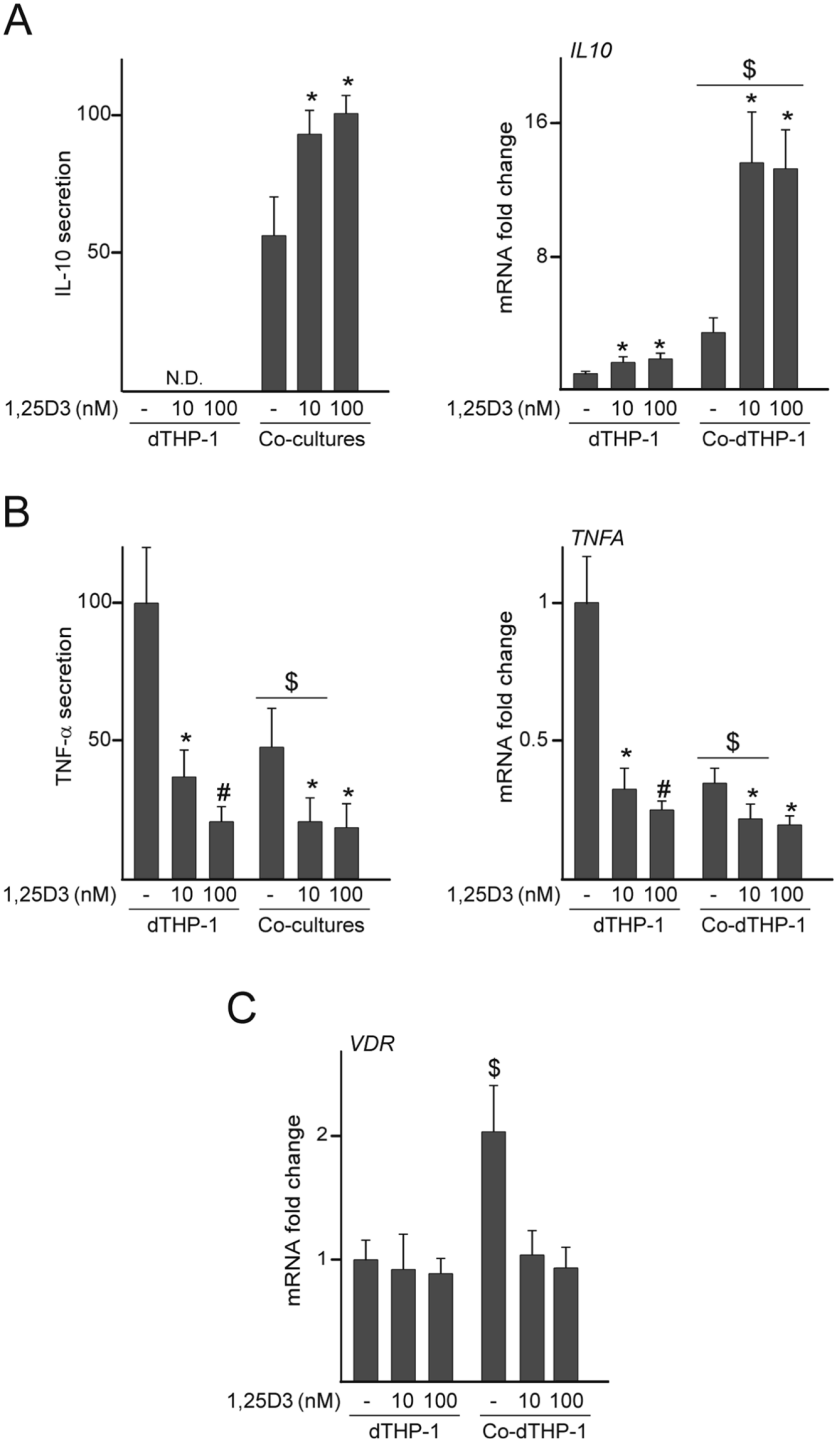
IL-10, TNF-α and VDR levels in co-cultures treated with 1,25D3. dTHP-1 cultured in isolation or co-cultured with MSC (Co-dTHP-1) were treated with 10 or 100 nM 1,25D3 or vehicle (−) for 72 h. IL-10 (**A**) and TNF-α (**B**) secretion (left panels) and mRNA fold changes (right panels). In these experiments, a relative secretion value of 100 corresponded to approximately 20 ± 0.8 pg of IL-10 and 307 ± 61 pg of TNF-α per ml of culture medium. *VDR* mRNA fold changes in dTHP-1 and Co-dTHP-1 (**C**). mRNA data are relative to those measured in untreated cells cultured in isolation, which were given the arbitrary value of 1. *p < 0.05 compared with untreated conditions; ^#^p < 0.05 compared with 10 nM 1,25D3; ^$^p < 0.05 compared with cells cultured in isolation under the same experimental conditions. N.D.: Not detected.

**Figure 3:**
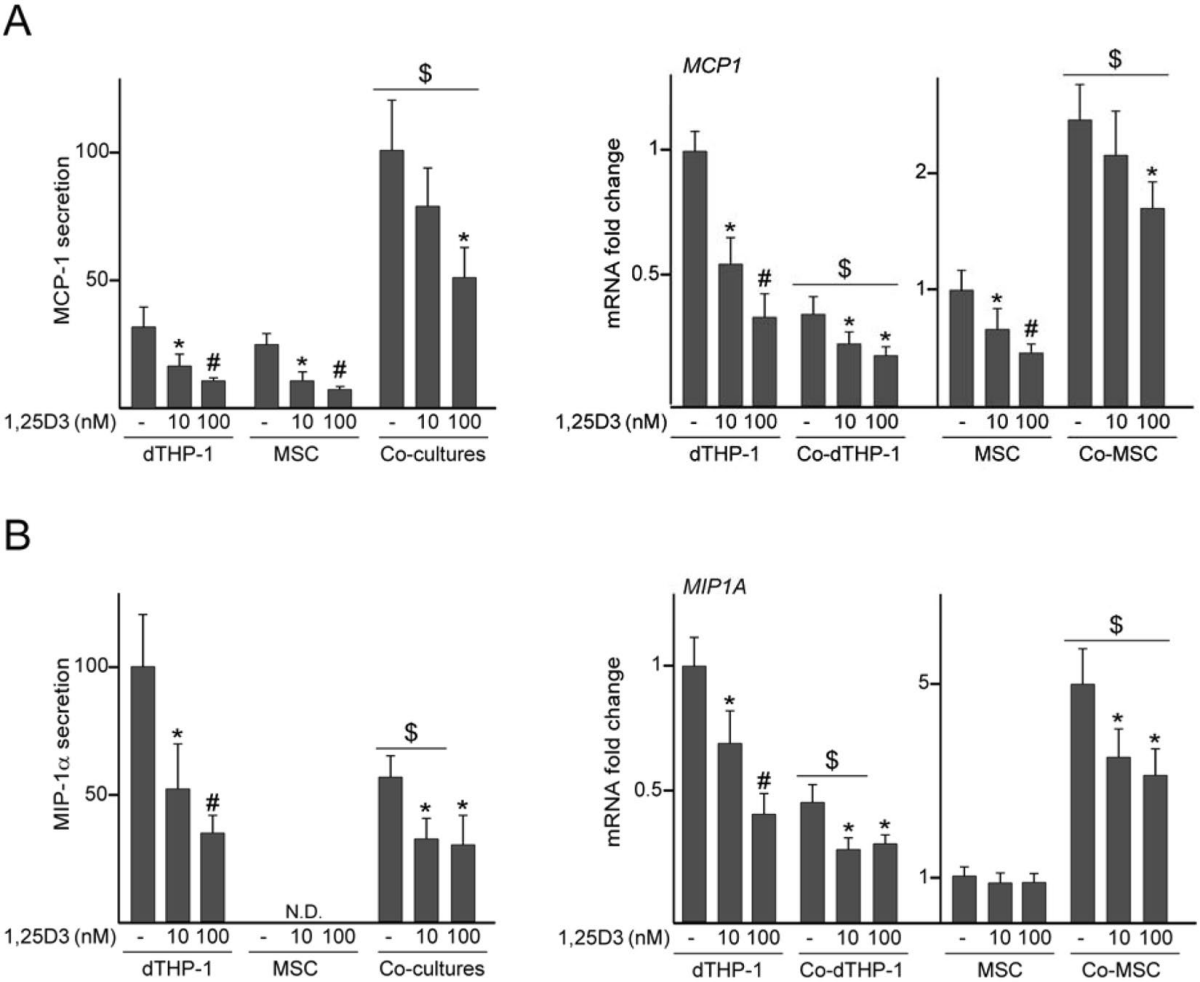
MCP-1 and MIP-1α levels in co-cultures treated with 1,25D3. dTHP-1 and MSC cultured in isolation or co-cultured (Co-dTHP-1 and Co-MSC, respectively) were treated with 10 or 100 nM 1,25D3 or vehicle (−) for 72 h. MCP-1 (**A**) and MIP-1α (**B**) secretion in cells cultured in isolation or co-cultures (left panels) and mRNA fold changes in each cell population (right panels). In these experiments, a relative secretion value of 100 corresponded to approximately 6.9 ± 1.5 ng of MCP-1 and 282 ± 56 pg of MIP-1α per ml of culture medium. mRNA data are relative to those measured in untreated cells cultured in isolation, which were given the arbitrary value of 1. *p < 0.05 compared with untreated conditions; ^#^p < 0.05 compared with 10 nM 1,25D3; ^$^p < 0.05 compared with cells cultured in isolation at the same experimental condition. N.D.: Not detected.

We next investigated whether 1,25D3 modulates the inflammatory state of dTHP-1 when co-cultured with MSC. Co-culturing led to an increase in the levels of the anti-inflammatory cytokine IL-10 and a decrease in TNF-α secretion (Fig. 2A,B). These changes were accompanied by an increase in *VDR* mRNA levels in dTHP-1 co-cultured with MSC (Fig. 2C). *IL10* mRNA levels in dTHP-1 cultured in isolation were up-regulated by 1,25D3, although the protein was undetectable in culture media (Fig. 2A). IL-10 protein and mRNA levels increased approximately 2- and 4-fold, respectively, in co-cultures exposed to 1,25D3 (Fig. 2A). As observed in cells cultured in isolation, 1,25D3 treatment of co-cultures decreased both protein and mRNA levels of TNF-α (Fig. 2B). We also examined the effect of 1,25D3 on the secretion of MCP-1 and macrophage inflammatory protein-1α (MIP-1α), chemokines involved in trauma-induced inflammation^21^. Treatment with 1,25D3 led to a dose-dependent decrease in the secretion and mRNA levels of the MCP-1 chemokine in dTHP-1 and MSC cultured in isolation (Fig. 3A). Co-culturing induced a substantial increase in MCP-1 concentration in media and mRNA levels in MSC (Fig. 3A). Treatment of co-cultures with the higher dose of 1,25D3 decreased MCP-1 secretion, which was accompanied by a reduction in *MCP1* mRNA levels in the stromal cells (Fig. 3A). *MCP1* expression in dTHP-1 decreased after co-culturing and further decreased after 1,25D3 treatment (Fig. 3A, right panel). The addition of 1,25D3 to dTHP-1 decreased MIP-1α at both the protein and mRNA levels in a dose-dependent manner (Fig. 3B). MIP-1α concentration in media decreased after co-culturing and further decreased following 1,25D3 exposure (Fig. 3B, left panel). These changes paralleled a reduction in *MIP1A* mRNA levels in co-cultured dTHP-1 and MSC after treatment with the hormone (Fig. 3B, right panel).

**Figure 4:**
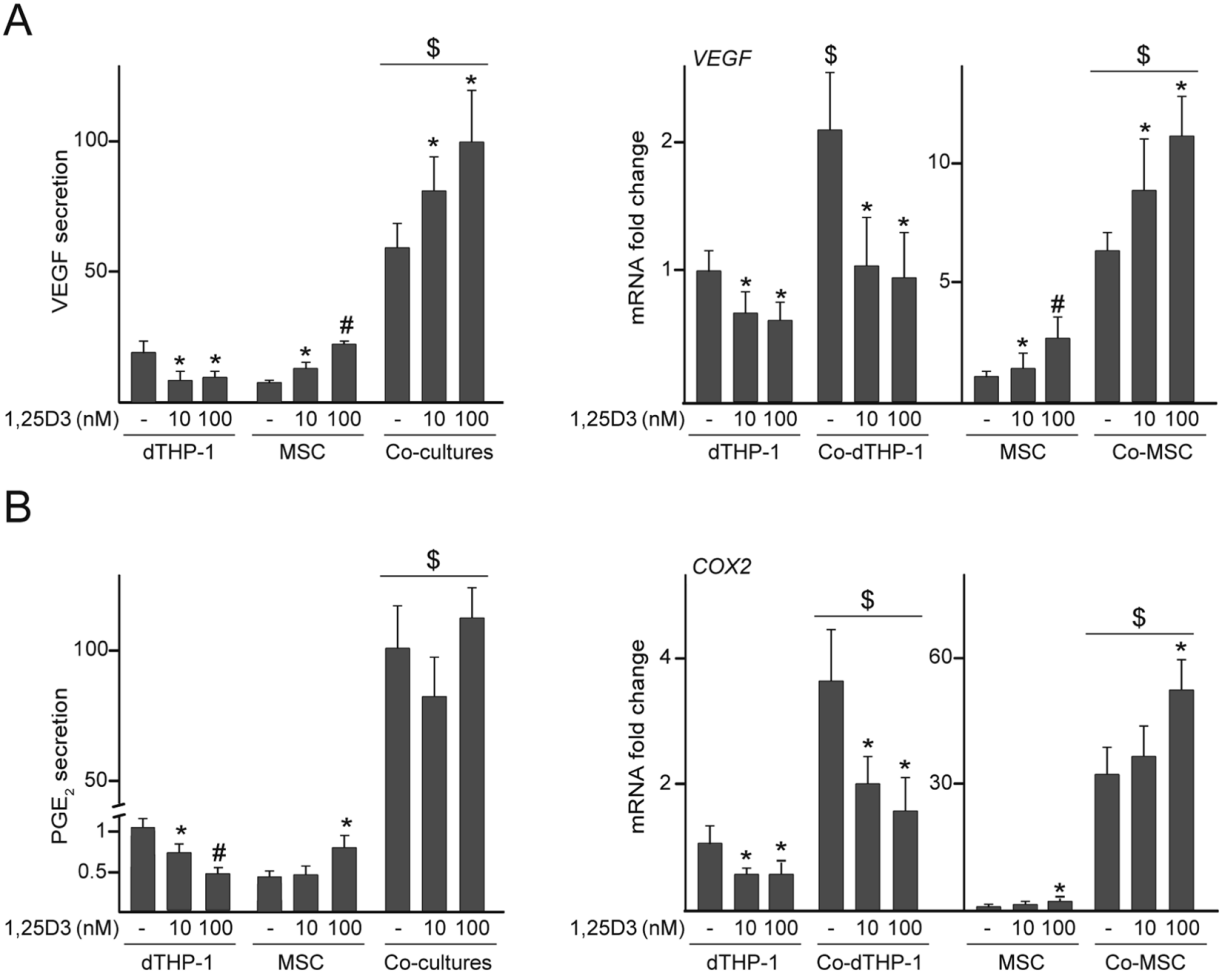
VEGF and PGE_2_ levels in co-cultures treated with 1,25D3. dTHP-1 and MSC cultured in isolation or co-cultured (Co-dTHP-1 and Co-MSC, respectively) were treated with 10 or 100 nM 1,25D3 or vehicle (−). VEGF (**A**) and PGE_2_ (**B**) secretion in cells cultured in isolation or co-cultures (left panels) and *VEGF* and *COX2* mRNA fold changes in each cell population (right panels). In these experiments, a relative secretion value of 100 corresponded to approximately 3.2 ± 0.7 pg of VEGF and 10 ± 2 ng of PGE_2_ per ml of culture medium. mRNA data are relative to those measured in untreated cells cultured in isolation, which were given the arbitrary value of 1. *p < 0.05 compared with untreated conditions; ^#^p < 0.05 compared with 10 nM 1,25D3; ^$^p < 0.05 compared with cells cultured in isolation under the same experimental conditions.

Having observed that 1,25D3 reduces the secretion of inflammatory factors in co-cultures of MSC and dTHP-1, we next investigated the influence of 1,25D3 on the secretion of vascular endothelial growth factor (VEGF) and prostaglandin E_2_ (PGE_2_), soluble mediators involved in MSC-mediated immunoregulation and tissue repair. Treatment with 1,25D3 enhanced the secretion of VEGF and PGE_2_ in MSC whereas the effect was opposite in dTHP-1 (Fig. 4A,B, left panels). Transcript levels of *VEGF* and cyclooxygenase-2 (*COX2)*, a key enzyme in PGE_2_ synthesis, followed a similar pattern to the secretion profiles of VEGF and PGE_2_ (Fig. 4A,B, right panels). Co-culturing dTHP-1 and MSC substantially increased the concentration of these factors in media as well as *VEGF* and *COX2* mRNA levels in both cell types. 1,25D3 enhanced VEGF secretion to the co-culture media, which correlated with an increase in *VEGF* expression at the mRNA level in MSC (Fig. 4A). *VEGF* expression in single-cultured or co-cultured dTHP-1 diminished after 1,25D3 exposure (Fig. 4A, right panel). As observed in single-cultured cells, 1,25D3 regulated *COX2* mRNA levels in co-cultured dTHP-1 and MSC in opposite trends (Fig. 4B, right panel). However, PGE_2_ concentration in co-culture media was not affected by 1,25D3 treatment (Fig. 4B, left panel).

### Effect of 1,25D3 on MSC osteogenesis and levels of bone-remodeling factors in MSC/dTHP-1 co-cultures

**Figure 5:**
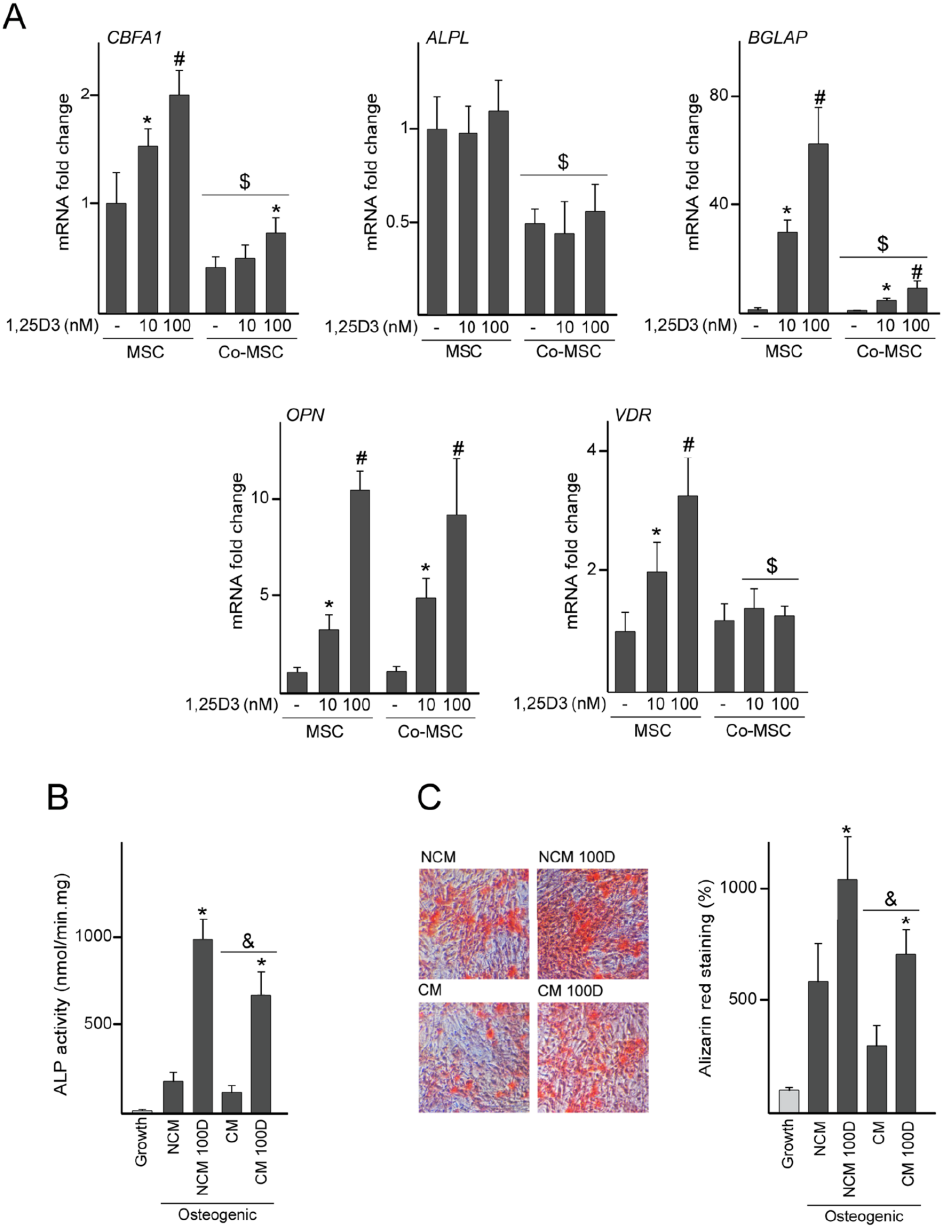
Effect of 1,25D3 on osteogenic differentiation of MSC exposed to dTHP-1. MSC cultured in isolation or co-cultured with dTHP-1 (Co-MSC) were treated with 10 or 100 nM 1,25D3 or vehicle (−) for 72 h. mRNA fold changes of the indicated genes in MSC and Co-MSC (**A**). Data are relative to those measured in untreated cells cultured in isolation, which were given the arbitrary value of 1. ALP activity (**B**) and alizarin red staining and quantification (**C**) in layers of MSC cultured under osteogenic conditions for 7 days or 14 days, respectively, in non-conditioned media (NCM) or in conditioned media from co-cultures (CM) treated with 100 nM 1,25D3 (100D) or vehicle. As controls, cells were cultured in the absence of osteogenic inductors (Growth). Quantitative data in C are relative to those measured in cells cultured in growth medium, which were given the arbitrary value of 100. *p < 0.05 compared with untreated conditions; ^#^p < 0.05 compared with 10 nM 1,25D3; ^$^p < 0.05 compared with cells cultured in isolation under the same experimental conditions; ^&^p < 0.05 compared with NCM under the same experimental conditions.

Inflammatory factors can interfere with the *in vitro* osteogenic capacity of MSC^22,23^. In fact, the expression at the mRNA level of the osteogenic differentiation markers core binding factor alpha-1 (*CBFA1*), alkaline phosphatase (*ALPL*) and *BGLAP* in MSC decreased by 2-fold after co-culturing with dTHP-1 (Fig. 5A). Given 1,25D3 reduces the secretion of inflammatory factors in co-cultures, we examined whether the hormone could enhance the osteogenic potential of co-cultured MSC. *CBFA1* expression in co-cultured MSC increased after 1,25D3 treatment, although this effect was lower than in isolated MSC (Fig. 5A). 1,25D3 did not affect *ALPL* mRNA levels in any condition (Fig. 5A). As observed for the *BGLAP* gene, exposure of MSC to 1,25D3 greatly increased transcript levels of the gene encoding for osteopontin (*OPN*) in a dose-dependent manner (Fig. 5A). 1,25D3-mediated increase of *BGLAP* mRNA levels was attenuated in co-cultures, whereas this effect was not observed for *OPN* (Fig. 5A). Although co-culturing did not affect the stromal expression of *VDR*, it prevented its dose-dependent increase in co-cultured MSC treated with 1,25D3 (Fig. 5A). To examine whether the reduction in *CBFA1*, *ALPL* and *BGLAP* expression in co-cultured MSC influences their osteogenesis ability, MSC were co-cultured with dTHP-1 for 72 h and then stimulated to undergo osteogenic differentiation in the absence of dTHP-1 for up to 14 days. Co-culturing MSC with dTHP-1 prior to osteogenesis induction did not influence their ability to develop mineralized matrix, as assessed by quantification of ALP activity and alizarin red staining (Supplementary Fig. S1). MSC co-cultured or not with 1,25D3 exhibited similar responses (Supplementary Fig. S1). We next studied whether continuous exposure to CM from co-cultures affects MSC osteogenesis. MSC cultured under osteogenic conditions with CM from co-cultures showed decreased ALP activity (Fig. 5B) and alizarin red staining (Fig. 5C) compared with cells cultured in non-conditioned media. However, MSC that received CM from co-cultures treated with 1,25D3 increased matrix maturation and mineralization.

**Figure 6:**
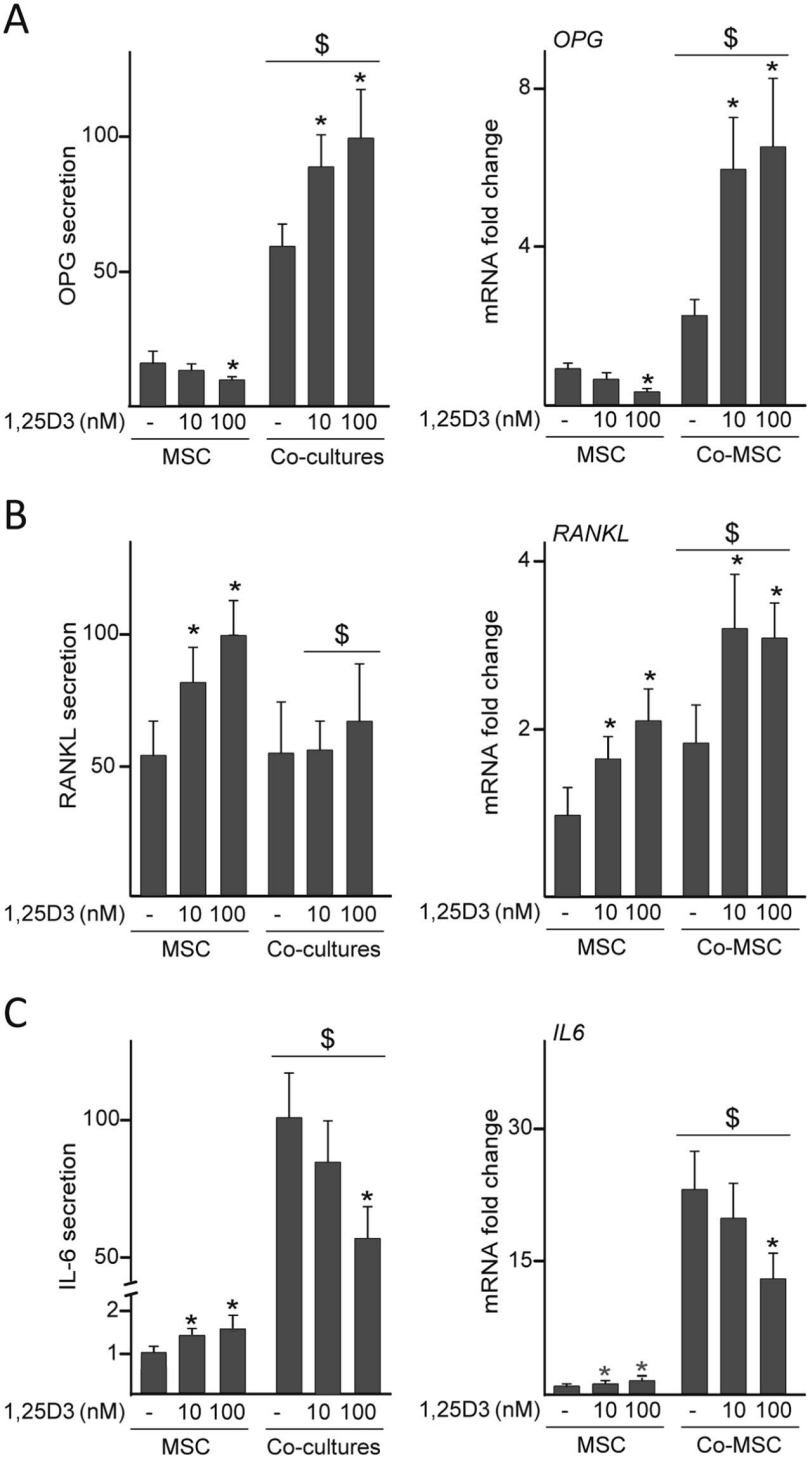
OPG, RANKL and IL-6 levels in co-cultures treated with 1,25D3. MSC cultured in isolation or co-cultured with dTHP-1 (Co-MSC) were treated with 10 or 100 nM 1,25D3 or vehicle (−) for 72 h. OPG (**A**), RANKL (**B**) and IL-6 (**C**) secretion (left panels) and mRNA fold changes (right panels). In these experiments, a relative secretion value of 100 corresponded to approximately 1.5 ± 0.3 ng of OPG, 38 ± 8 pg of RANKL and 70 ± 17 ng of IL-6 per ml of culture medium. mRNA data are relative to those measured in untreated cells cultured in isolation, which were given the arbitrary value of 1. *p < 0.05 compared with untreated conditions; ^$^p < 0.05 compared with cells cultured in isolation under the same experimental conditions.

In addition to their ability to differentiate into functional osteoblasts, MSC secrete osteoprotegerin (OPG) and RANKL, important regulators of bone remodeling and targets in the skeletal action of 1,25D3. We next investigated the effect of 1,25D3 on these soluble mediators in MSC cultured in isolation or co-cultured. Treatment of MSC with 100 nM 1,25D3 decreased OPG secretion and mRNA levels, whereas no effects were detected when cells were treated with 10 nM 1,25D3 (Fig. 6A). Treatments with both doses similarly increased levels of free RANKL in media and the relative amount of *RANKL* transcripts (Fig. 6B). OPG secretion highly increased after co-culturing whereas the concentration of RANKL in media was similar in MSC cultured in isolation and in co-cultures (Fig. 6A,B, left panels). In contrast to that observed in single-cultured cells, both doses of 1,25D3 increased OPG secretion in co-cultures, whereas they did not have any effect on RANKL levels (Fig. 6A,B, left panels). Changes in OPG secretion induced by 1,25D3 treatment paralleled changes at the mRNA level (Fig. 6A, right panel). *RANKL* mRNA levels were higher in MSC co-cultured with dTHP-1 than in MSC cultured in isolation, and were further increased after 1,25D3 exposure (Fig. 6B, right panel). Treatments of MSC with both doses similarly increased IL-6 levels in media (Fig. 6C, left panel). In contrast, levels of this cytokine, highly induced by co-culturing MSC with dTHP-1, did not experience any significant change after exposure of co-cultures to 10 nM 1,25D3 and decreased after treatment with the higher dose. Regulation of *IL6* mRNA levels by 1,25D3 correlated with the observed changes in IL-6 concentration in media (Fig. 6C, right panel).

### Effect of 1,25D3 and topographical cues on secreted factors by MSC/dTHP-1 co-cultures and MSC osteogenesis

**Figure 7:**
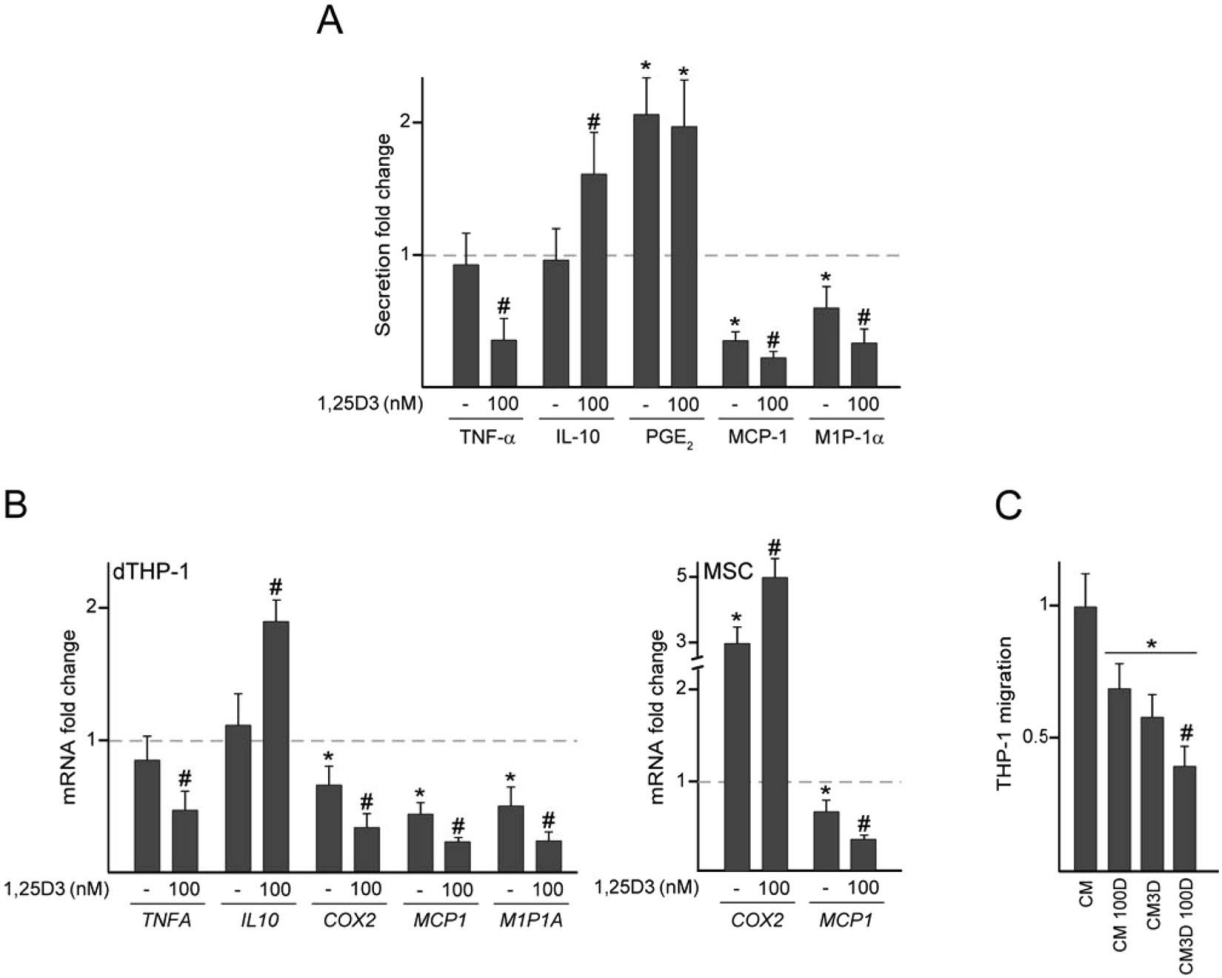
Effect of 1,25D3 on inflammatory factors in 3D co-cultures and on monocyte migration. dTHP-1 co-cultured with MSC seeded in 3D substrates were treated with 100 nM 1,25D3 or vehicle (−) for 72 h. Secretion levels of the indicated soluble factors in 3D co-cultures (**A**). mRNA fold changes of the indicated genes in co-cultured dTHP-1 or MSC (**B**). Data are relative to the protein or mRNA values determined in untreated 2D co-cultures, which were given the arbitrary value of 1. Migration capacity of THP-1 cells toward conditioned media from 2D co-cultures (CM) or 3D co-cultures (CM3D) treated with 100 nM 1,25D3 (100D) or vehicle (**C**). In these experiments, a relative value of 100 corresponded to approximately 75 ± 17 ×  10^3^ migrated cells. *p < 0.05 compared with untreated 2D co-cultures or CM; ^#^p < 0.05 compared with untreated 3D co-cultures or CM3D.

We recently reported that the architecture of the substrates that harbor MSC decisively influences their crosstalk with macrophages^16^. Using the same 3D substrates, we herein investigated whether 1,25D3 affected the secretion profile of soluble factors involved in inflammation, angiogenesis and bone remodeling in co-cultures of dTHP-1 and 3D-arranged MSC (3D co-cultures). Co-cultures with MSC seeded directly in flat transwells (conventional/2D co-cultures) were used for comparative purposes. TNF-α and IL-10 levels in co-cultures were not affected by seeding MSC in 3D substrates (Fig. 7A). The effect of 1,25D3 on these inflammatory factors in 3D co-cultures was similar to that observed in 2D co-cultures, decreasing the secretion of TNF-α while conversely stimulating the production of IL-10. Changes in TNF-α and IL-10 secretion induced by 1,25D3 treatment paralleled changes in mRNA levels in co-cultured dTHP-1 (Fig. 7B, left panel). Compared with 2D co-cultures, 3D disposition of MSC co-cultured with dTHP-1 induced an increase in PGE_2_ levels while decreasing MCP-1 and MIP-1α secretion (Fig. 7A). PGE_2_ production in 3D co-cultures was not affected by 1,25D3 treatment (Fig. 7A), although *COX2* mRNA levels were subjected to differential modulation in each cell type, decreasing in co-cultured dTHP-1 and increasing in co-cultured MSC (Fig. 7B). Exposure of 3D co-cultures to 1,25D3 decreased MCP-1 and MIP-1α levels as well as their mRNA levels in co-cultured dTHP-1 (Fig. 7A,B). Treatment with 1,25D3 also decreased *MCP1* mRNA levels in 3D-arranged MSC co-cultured with dTHP-1 (Fig. 7B, right panel). The levels of MCP-1 and MIP-1α in 1,25D3-treated 3D co-cultures were about 2-fold lower than in 1,25D3-treated 2D co-cultures (Figs 3 and 7). Given both chemokines are involved in the recruitment of monocytes, we investigated whether 1,25D3 treatment of co-cultures influenced THP-1 migration by using CM. Compared with untreated co-cultures, THP-1 recruitment was reduced when CM from co-cultures treated with 1,25D3 were employed as a stimulus (Fig. 7C). THP-1 migration induced by CM of 1,25D3-treated 3D co-cultures was lower than that induced by CM of 2D co-cultures exposed to the hormone.

**Figure 8:**
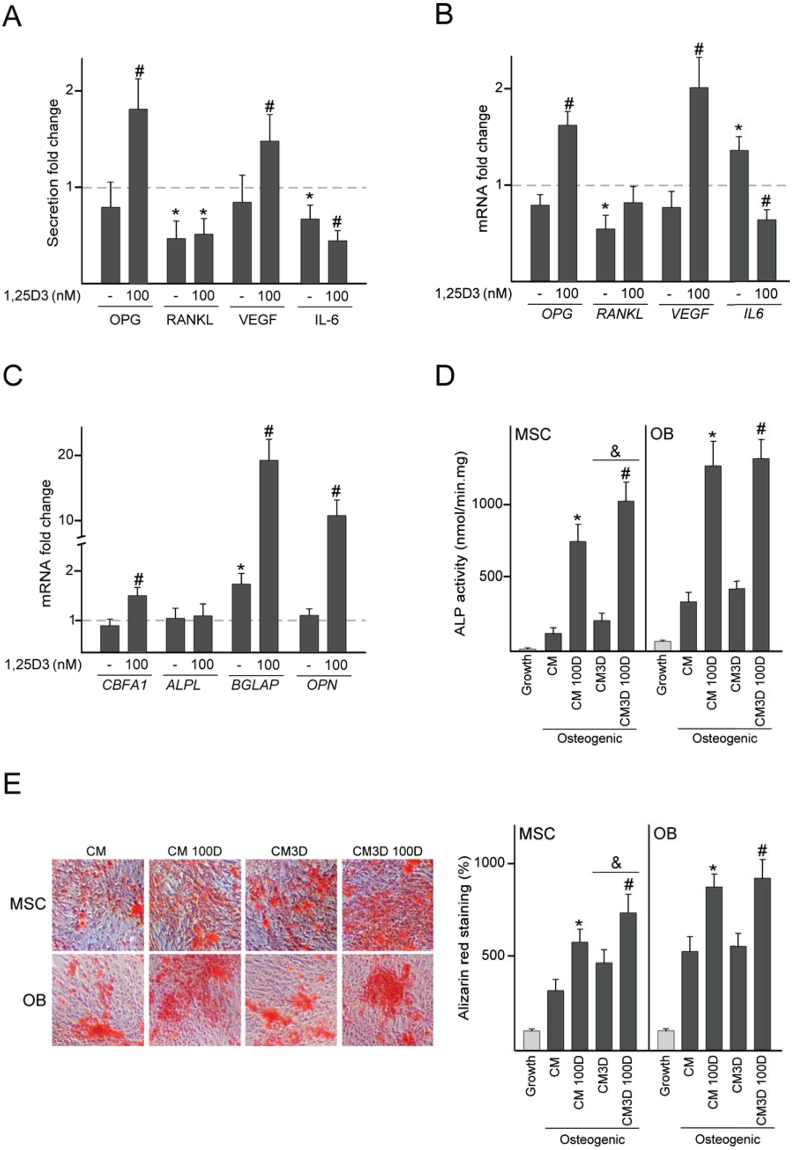
Effect of 1,25D3 on bone-related factors in 3D co-cultures and on osteogenic differentiation. dTHP-1 co-cultured with MSC seeded in 3D substrates were treated with 100 nM 1,25D3 or vehicle (−) for 72 h. Secretion levels of the indicated soluble factors in 3D co-cultures (**A**). mRNA fold changes of the indicated genes in co-cultured MSC seeded in 3D substrates (**B**,**C**). Data are relative to the protein or mRNA values determined in untreated 2D co-cultures, which were given the arbitrary value of 1. ALP activity (**D**) and alizarin red staining and quantification (**E**) in layers of MSC or osteoblasts (OB) cultured under osteogenic conditions for 7 days or 14 days, respectively, in conditioned media from 2D co-cultures (CM) or 3D co-cultures (CM3D) treated with 100 nM 1,25D3 (100D) or vehicle. As controls, cells were cultured in the absence of osteogenic inductors (Growth). Quantitative data in **E** are relative to those measured in cells cultured in growth medium, which were given the arbitrary value of 100. *p < 0.05 compared with untreated 2D co-cultures or CM; ^#^p < 0.05 compared with untreated 3D co-cultures or CM3D; ^&^p < 0.05 compared with CM or CM 100D under the same experimental conditions.

Figure 8A shows that 3D arrangement of MSC co-cultured with dTHP-1 led to a decrease in the concentration of the pro-resorptive factors RANKL and IL-6. Treatment of 3D co-cultures with 1,25D3 further decreased IL-6 secretion. OPG and VEGF levels, which were similar in both 3D and 2D co-cultures, increased after 1,25D3 treatment. These changes in protein secretion induced by 1,25D3 paralleled changes in *OPG* and *VEGF* mRNA levels in co-cultured MSC (Fig. 8A,B). Finally, we studied whether topographical cues in combination with 1,25D3 treatment could modulate osteogenic differentiation of MSC when co-cultured with dTHP-1. MSC seeded in 3D substrates and co-cultured with dTHP-1 expressed higher *BGLAP* mRNA levels than MSC that had been co-cultured in 2D substrates (Fig. 8C). No changes were observed in *CBFA1*, *ALPL* and *OPN* mRNA levels. When MSC co-cultured in 3D substrates were induced to undergo osteogenic differentiation in the absence of dTHP-1, matrix mineralization was similar to that of MSC that had been co-cultured in 2D substrates (Supplementary Fig. S2). However, experiments using CM revealed that continuous exposure to secreted factors from 3D co-cultures resulted in higher ALP activity and alizarin red staining than exposure to CM from 2D co-cultures (Fig. 8D,E). MSC incubated with CM from 1,25D3-treated co-cultures increased matrix maturation and mineralization, an effect more pronounced with CM from 3D than 2D co-cultures (Fig. 8D,E). We also conducted experiments to evaluate the effect of continuous exposure to CM from co-cultures on the functionality of primary osteoblasts isolated from trabecular bone. As observed for MSC, osteoblasts proliferated at the same rate when incubated in CM from co-cultures treated with 1,25D3 or vehicle (Supplementary Fig. S3). ALP activity and alizarin red staining increased when osteoblasts were incubated in CM from co-cultures treated with 1,25D3, but no differences were found between CM from 2D and 3D co-cultures (Fig. 8D,E).

## Discussion

A positive correlation between vitamin D supplementation and bone healing has been observed in studies conducted with animal models, in which the hormone stimulated fracture repair and increased the mechanical strength of the callus^24–26^. Vitamin D supplementation during the early healing stage promoted new bone formation and increased bone density in mandibular sockets of dogs implanted with biphasic calcium phosphate ceramic^27,28^. The pro-osteogenic effect of vitamin D has been further substantiated by *in vitro* and *in vivo* studies using titanium implants coated with 1,25D3 precursor^29,30^. In addition to regulating osteoblast functionality, 1,25D3 has been shown to reduce the secretion of inflammatory factors by macrophages, which interact with osteoblast lineage cells during bone healing^10,31^. The present study provides insight into the mechanisms by which 1,25D3 might accelerate the bone regeneration process through the regulation of paracrine communications between MSC and macrophages. In addition, data herein highlight the importance of paracrine factors in the modulation of the biological actions of 1,25D3, given we have observed differential, and even opposing, effects of the hormone in single cultures of MSC or macrophages and co-cultures of both cell types.

Many studies have demonstrated that MSC can regulate the activation and function of macrophages through the secretion of soluble factors. We previously reported that MSC display immunomodulatory effects on dTHP-1, given co-culturing both cell types reduced TNF-α levels and increased IL-10 production. These changes in protein secretion correlated with increased levels of PGE_2,_ MCP-1 and VEGF in the co-culture media^16^. To our knowledge, the effect of 1,25D3 on the secretion of soluble mediators involved in MSC-mediated immunoregulation has not been explored to date. In this study, we detected that 1,25D3 activates MSC to produce PGE_2_, suggesting that this hormone might be potentiating their immunomodulatory actions. In addition, levels of the pro-angiogenic factor VEGF increase after treatment of MSC with the hormone, as previously observed in endothelial progenitor cells^32^. Co-culture experiments demonstrated that 1,25D3 could regulate the paracrine interactions established between MSC and macrophages by modulating the production of some of these soluble factors. 1,25D3 promoted the switch of co-cultured macrophages toward an anti-inflammatory phenotype because it increased *IL10* expression while having the opposite effect on *TNFA*. VEGF levels in co-cultures were induced by treatment with the hormone, which likely reflects 1,25D3 enhancement of the angiogenic potential of MSC. As observed in isolated cultures of MSC and dTHP-1, 1,25D3 also decreased MCP-1 levels after co-culturing both cell types. However, 1,25D3 failed to modulate PGE_2_ levels in co-cultures, although cells cultured in isolation were sensitive to the hormone. These data highlight the importance of paracrine factors in understanding the effects of 1,25D3 on a particular cell type and emphasize the need for cautious extrapolation of data obtained in single-culture models. It is worth noting that dTHP-1 constitutively expresses *VDR*, but its mRNA and protein levels were not regulated by 1,25D3, as observed in human peripheral blood macrophages^33^. *VDR* mRNA levels in dTHP-1 increased after co-culturing with MSC, suggesting that soluble factors produced in co-cultures might activate signaling cascades that lead to the regulation of the transcript levels. In this regard, the stress-activated protein kinases p38 and JNK can stimulate *VDR* promoter activity in human breast cancer cells, independently of 1,25D3^34^. Curiously, up-regulation of *VDR* mRNA levels in dTHP-1 co-cultured with MSC was prevented by 1,25D3, emphasizing the role of the inflammatory milieu in the adjustment of *VDR* expression by the hormone.

In contrast to dTHP-1, *VDR* expression in MSC was regulated positively by the hormone. 1,25D3 strongly regulated the stromal expression of the osteogenic markers *BGLAP* and *OPN*, which contain functional VDRE in their promoters^20,35^. *CBFA1* expression was also stimulated by 1,25D3, an effect previously observed in human primary osteoblasts^36^. Inflammatory factors released upon injury, such as TNF-α, IL-6 and PGE_2_, can inhibit the expression of bone-specific genes at the transcriptional level and compromise MSC osteogenic differentiation^22,23,37,38^. In addition, TNF-α has been shown to induce osteoblast resistance to 1,25D3^39^. In keeping with this evidence, data herein show that co-culture with dTHP-1 decreases *CBFA1, ALPL* and *BGLAP* mRNA levels in MSC. In addition, mRNA accumulation of *VDR, CBFA1* and *BGLAP* after 1,25D3 treatment was lower in co-cultured than in isolated MSC. Although co-culturing for 72 h decreased the mRNA levels of these osteogenic markers in MSC, it did not influence their ability to develop mineralized matrix when further cultured in the absence of dTHP-1. In this regard, a recent study showed that an exposure time of at least 7 days was required for PGE_2_ to elicit an inhibitory effect on mineralized matrix formation by MSC^23^. Our experiments using CM indicated that continuous exposure to soluble factors secreted by co-cultures interferes with MSC osteogenic potential. Interestingly, incubation with CM from co-cultures treated with 1,25D3 stimulated matrix maturation and mineralization. This effect can be attributed to the capacity of 1,25D3 to induce *in vitro* osteoblast differentiation^6,40^, but also to the lowered inflammatory factors in CM from treated co-cultures.

In addition to direct effects on osteoblast phenotypic gene expression, 1,25D3 regulates osteoclast differentiation indirectly by acting on osteoblasts. Supporting the notion that osteoprogenitors and osteoblasts respond to 1,25D3 in a potentially pro-osteoclastogenic manner^41^, we found that 1,25D3 increased levels of free RANKL in MSC culture media, which correlated with a decrease in the production of OPG, the decoy receptor for RANKL. A previous study reported that treatment of MSC with TNF-α increased OPG expression and that co-culture of TNF-α-stimulated MSC and monocytes decreased osteoclast activation, compared with unstimulated MSC^42^. This suggests that MSC might suppress osteoclastogenesis during inflammation. In this regard, data herein indicate that OPG production substantially increased in co-cultures of MSC and dTHP-1, whereas levels of free RANKL remained unchanged. Interestingly, 1,25D3 effects on these regulators of bone remodeling were different in single-cultured or co-cultured MSC. Thus, treatment of co-cultures with 1,25D3 increased OPG expression, in contrast to that observed in single-cultured MSC. In addition, co-culturing MSC with dTHP-1 prevented 1,25D3-induced increase of RANKL levels. The effect of 1,25D3 on IL-6, which amplifies pro-resorptive signals, was also dependent on the microenvironment, given its levels decreased in co-cultures after exposure to the hormone, whereas it increased in single cultures of MSC. These data indicate that paracrine factors play an important role in regulating the biological effects of 1,25D3 on osteoprogenitors and suggest that 1,25D3 might act at the onset of inflammation, tilting the balance between bone resorption and formation toward the latter.

Tailoring surface properties of biomaterials is a promising strategy to modulate the host response for tissue regeneration applications^43^. We have shown in a previous study that microporous surfaces activated MSC for enhanced immunomodulation^16^. Using the same substrates, we observed herein that 1,25D3 treatment increases IL-10, VEGF and OPG production in 3D co-cultures, suggesting that 1,25D3 might lead to a reduction of the inflammatory response in favor of tissue repair. Compared with 2D co-cultures, 3D arrangement of MSC co-cultured with macrophages leads to an important decrease in IL-6, MCP-1 and MIP-1α secretion. Treatment of 3D co-cultures with 1,25D3 further reduced the levels of these inflammatory mediators, reaching values lower than in 2D co-cultures exposed to the hormone. The decrease in MCP-1 and MIP-1α chemokine levels induced by 3D arrangement of MSC and 1,25D3 in co-cultures paralleled a reduction in the amount of THP-1 migration toward CM. Given osteoclasts derive from monocyte progenitors and are activated by RANKL, reduction in monocyte migration promoted by 1,25D3, along with the decrease in RANKL induced by 3D substrates, might contribute to diminished bone resorption, leading to a positive bone balance. The osteogenic potential of MSC cultured with CM from 3D co-cultures was higher than that of cells cultured with CM from 2D co-cultures, which must be related to the lower concentration of pro-inflammatory factors, such as IL-6, MCP-1 and MIP-1α in 3D co-cultures. In fact, there are reports showing that the deleterious effects of inflammatory factors on osteoblast differentiation from MSC are dose-dependent^22,23^. Induction of matrix mineralization was similar in primary osteoblasts incubated in CM from 2D or 3D co-cultures, suggesting that inflammatory factors modulate the osteogenic differentiation process of undifferentiated cells but do not affect the functionality of terminally differentiated osteoblasts. As expected, we observed that CM from co-cultures containing 1,25D3 stimulated ALP activity and matrix calcification. The increase in osteogenic differentiation of MSC associated with 1,25D3 treatment was higher after incubation with CM from 3D co-cultures than with CM from 2D co-cultures. These results suggest that topographical cues and 1,25D3 might act in concert to accelerate osteoprogenitor commitment by regulating paracrine interactions with macrophage-lineage cells.

In summary, our results indicate that 1,25D3 reduces pro-inflammatory cytokine production in co-cultures of MSC and macrophages and promotes MSC osteogenic differentiation in an inflammatory milieu. Current efforts to minimize host reaction following implantation of a biomaterial device include tuning the biomaterial surface as well as delivery of anti-inflammatory drugs from the implanted device^43–45^. Future studies will investigate whether manufacturing of 3D substrates that provide topographical cues and local delivery of 1,25D3 might promote an ideal balance of soluble mediators for the restoration of functional bone.

## Methods

### Cell culture

Purified human bone marrow-derived MSC were purchased from Lonza (Basel, Switzerland) and expanded in a defined medium (Lonza) consisting of basal medium and Single Quots growth supplements containing fetal bovine serum (FBS) and antibiotics. All the experiments were performed below the seventh cell passage using six different batches of MSC isolated from donors aged 18–31 years. Human acute monocytic leukemia THP-1 cells (ECACC, Salisbury, Wiltshire, UK) were grown in RPMI-1640 medium (Lonza) containing 10% (v/v) FBS and antibiotics. To induce macrophage differentiation, THP-1 cells were treated with 100 ng/ml TPA (Sigma, Madrid, Spain) for 12 h, washed with phosphate-buffered saline (PBS) and further incubated in fresh medium for 24 h^16^. Human osteoblasts were obtained from trabecular bone explants aseptically collected from patients undergoing total knee replacement as previously described^46^. Patients enrolled in this research signed an informed consent and all the procedures using human tissue designated “surgical waste” were approved by the Ethics Committee for Clinical Research at La Paz University Hospital (Date of Approval: 20/05/2016). All the experiments and methods were performed in accordance with relevant guidelines and regulations. Each bone sample was processed separately and experiments were performed using independent cultures obtained from six patients aged 62–70 years. Experiments were performed with osteoblasts cultured up to the second passage. Bone fragments were cultured in growth medium consisting of Dulbecco’s modified eagle’s medium (DMEM, Lonza) supplemented with 15% (v/v) FBS and antibiotics. Cells were maintained at 37 °C in a humidified 5% CO_2_ incubator.

Conventional/2D co-cultures of MSC and dTHP-1 were set up using a polyester membrane cell culture insert of 0.4 μm pore size (Corning, Lowell, MA) that allows humoral contact of both cell types in the absence of direct cell-to-cell contact. THP-1 cells were seeded at a density of 4 × 10^5^ cells/well into 6-well plates and treated with TPA, as described above. MSC were seeded at a density of 8 × 10^4^ cells in the inserts and cultured for 24 h. To set up the 3D co-cultures, MSC were seeded in highly porous polystyrene scaffolds (Alvetex®, Reinnervate, Durham, UK), which were placed into the inserts as previously described^16^, and co-cultured with dTHP-1. As controls, only dTHP-1 or MSC were cultured in wells or inserts, respectively. The 1,25D3 (Sigma) was dissolved at 10 mM in 100% ethanol. Once assembled, the co-cultures were incubated in 3 ml of a mix of equal volumes of RPMI and DMEM containing 12.5% FBS, and immediately after, treated with 10 or 100 nM 1,25D3 or vehicle. Based on our previous study on the modulation of the secretory profile of inflammatory markers in co-cultures, the effect of 1,25D3 on paracrine interactions between MSC and dTHP-1 was examined after treatment for 72 h^16^. A schematic illustration of the set up of co-cultures is shown in Supplementary Figure S4. Cell viability remained greater than 90% under all experimental conditions.

### Gene expression

Total RNA was isolated using TRI Reagent (Molecular Research Center, Inc., Cincinnati, OH, USA), according to the manufacturer’s instructions. Complementary DNAs were prepared from total RNA using the Transcriptor First Strand cDNA Synthesis Kit using an anchored-oligo (dT)_18_ primer (Roche Applied Science, Indianapolis, IN, USA). Real-time quantitative PCR was performed using LightCycler FastStart DNA Master SYBR Green I and LightCycler detector (both from Roche Applied Science). Quantitative expression values were extrapolated from standard curves, and normalized to β2-microglobulin (β2 M) values. Specific oligonucleotide primers are shown in Supplementary Table S1.

### Immunoenzymatic assays

Cell culture media were collected, filtered and centrifuged at 1200 g for 10 min. Media were supplemented with 2 µg/ml aprotinin, 17.5 µg/ml phenyl-methylsulfonyl fluoride, 1 µg/ml pepstatin A and 50 µg/ml bacitracin (all from Sigma) and frozen at −80 °C. Human specific ELISA kits were used to measure OPG (Bender MedSystems GmbH, Vienna, Austria), free soluble RANKL (Biomedica Gruppe, Vienna, Austria), MIP-1α (Biosensis, Thebarton, Australia) and PGE_2_ (Cayman Chemical Company, Ann Arbor, MI), according to the manufacturer’s instructions. The detection limits of the kits were 2.5 pg/ml for OPG, 1.5 pg/ml for RANKL, 10 pg/ml for MIP-1α and 15 pg/ml for PGE_2_. Levels of TNF-α, IL-6, IL-10 and MCP-1 and VEGF were determined using BD CBA Flex Sets (BD Biosciences, San Jose, CA), following the manufacturer’s instructions. The data were acquired using a FACSCalibur flow cytometer and analyzed with the FCAP Array Software version 3.0 (BD Biosciences). The detection limits of the CBA Flex Sets were 3.7 pg/ml for TNF-α, 2.5 pg/ml for IL-6, 3.3 pg/ml for IL-10, 1.3 pg/ml for MCP-1 and 4.5 pg/ml for VEGF.

### Immunofluorescence assays

Cells were seeded at a density of 2 × 10^4^ cells/well in 8-well chamber slides and treated with 100 nM 1,25D3 or vehicle. After 3 days in culture, cells were fixed with 4% (w/v) formaldehyde in PBS and permeabilized with 0.1% Triton X-100 in PBS. For immunostaining, cells were blocked in PBS containing 2% bovine serum albumin (BSA) and 0.05% Tween 20 and then stained with mouse anti-human Oct-4, Sox-2, Stro-1 Abs (all from Chemicon, Harrow, UK) and rabbit anti-human VDR Abs (Santa Cruz, Heidelberg, Germany) diluted 1:50 in 1% BSA in PBS. After washing with 0.05% Tween in PBS, cells were incubated with goat anti-mouse Alexa-Fluor 488 or goat anti-rabbit Alexa-Fluor 594 secondary Abs (Molecular Probes, Leiden, Netherlands) diluted 1:1000 (v/v) in PBS containing 1% BSA. After washing with 0.05% Tween in PBS, cells were examined using a confocal microscope (Leica TCS SPE, Leica Microsystems, Heidelberg, Germany).

### Flow cytometry assays

Immunofluorescence staining of cell surface antigens was performed by incubating cells for 30 min at 4 °C in the dark with mouse anti-human CD44-FITC, CD34-FITC, human leukocyte antigen (HLA)-DR-FITC, CD105-PE, CD14-PE, CD11b-PE, CD29-APC and CD45-APC Abs (all from BD Bioscience, San Jose, CA). Cells incubated in the absence of antibodies were used as negative controls. Cells were then washed 3 times with PBS, fixed with 1% (w/v) formaldehyde in PBS and subjected to flow cytometry using a FACSCalibur analyzer and CellQuest software (BD Biosciences).

### Migration assays

Migration assays were conducted using transwell inserts of 5 μm pore size (Corning) placed into 24-well plates. THP-1 cells were washed twice with PBS and resuspended in serum-free RPMI at a density of 1.5 × 10^6^ cells/ml. Then, 200 μl of cell suspension was added to the upper chamber and 500 μl of CM of co-cultures treated with 100 nM 1,25D3 or vehicle were added to the lower chamber. After 5 h of incubation, cells that migrated to the lower chamber were collected and counted, employing the trypan blue dye exclusion method. Nonspecific migration was evaluated by using culture medium without serum.

### ALP activity and cell layer calcification assays

Osteoblasts and MSC were seeded at a density of 1 × 10^4^ cells/well in 12-well plates and incubated in a mix of equal volumes of osteogenic medium and CM from co-cultures treated with 100 nM 1,25D3 or vehicle. A parallel set of cells were cultured in a mix containing media not conditioned by co-cultures (NCM). To determine MSC osteogenic differentiation after co-culturing, MSC were co-cultured with dTHP-1 for 72 h and then cultured in osteogenic medium in the absence of dTHP-1. Osteogenic medium consisted of growth medium supplemented with 3 × 10^−4^ M ascorbic acid, 10^−2^ M β-glycerophosphate and 10^−7^ M of dexamethasone (all from Sigma). Cells cultured in growth medium were used as control. Media were replaced every 3 days. After 7 days of culture, cell layers were extracted with 5 × 10^−2^ M Tris-HCl pH 8.0, 5 × 10^−1^ M NaCl and 1% Triton X-100 and supplemented with a mixture of protease inhibitors. ALP activity was assessed in cell layers by determining the release of p-nitrophenol from p-nitrophenyl phosphate (Sigma). The data were normalized to the total protein amounts determined by a Bradford-based protein assay (Bio-Rad Laboratories Inc., Hercules, CA), using BSA as protein standard. The degree of cell layer calcification was assessed in cells cultured for 14 days using alizarin red staining. Briefly, cells were fixed with ethanol and stained with 40 mM alizarin red in deionized water at pH 4.2. The bound stain was eluted with 10% (w/v) cetylpyridinium chloride and the absorbance at 562 nm was measured using a Synergy4 spectrofluorometer (BioTek Instruments, Winooski, VT).

### Statistical analysis

The statistical analyses were performed using the Statistical Program for Social Sciences version 11.5 (SPSS Inc, Chicago, IL, USA). Data are presented as means ± SD of at least five independent experiments. Two-way ANOVAs for repeated measures were performed to analyze data from co-culture experiments, factoring for culture conditions and treatments. One-way ANOVAs were used for experiments using conditioned media. Post hoc comparisons were analyzed by a 95% confidence interval adjusted by the Bonferroni method. The p-values < 0.05 were considered to be significant.

## Electronic supplementary material

Supplementary Information
