# Publisher Correction: Paracrine interactions between mesenchymal stem cells and macrophages are regulated by 1,25-dihydroxyvitamin D3

**DOI:** 10.1038/s41598-017-17989-5

**Published:** 2018-01-05

**Authors:** Laura Saldaña, Gema Vallés, Fátima Bensiamar, Francisco José Mancebo, Eduardo García-Rey, Nuria Vilaboa

**Affiliations:** 1 Hospital Universitario La Paz-IdiPAZ, Paseo de la Castellana 261, 28046 Madrid, Spain; 2 CIBER de Bioingeniería, Biomateriales y Nanomedicina (CIBER-BBN), Madrid, Spain

Correction to: *Scientific Reports* 10.1038/s41598-017-15217-8, published online 03 November 2017

The original HTML version of this Article contained a typographical error in the publication date ‘03 November 2017’ which was incorrectly given as ‘06 November 2017’. This has now been corrected in the HTML version of the Article.

