# Publisher Correction: Paracrine interactions between mesenchymal stem cells and macrophages are regulated by 1,25-dihydroxyvitamin D3

**DOI:** 10.1038/s41598-018-22377-8

**Published:** 2018-03-06

**Authors:** Laura Saldaña, Gema Vallés, Fátima Bensiamar, Francisco José Mancebo, Eduardo García-Rey, Nuria Vilaboa

**Affiliations:** 1 Hospital Universitario La Paz-IdiPAZ, Paseo de la Castellana 261, 28046 Madrid, Spain; 2 CIBER de Bioingeniería, Biomateriales y Nanomedicina (CIBER-BBN), Madrid, Spain

Correction to: *Scientific Reports* 10.1038/s41598-017-15217-8, published online 03 November 2017

An error occurred after publication resulting in the inadvertent omission of the Article and Volume numbers from the PDF version of this Article. This error has now been corrected in the PDF version of the Article; the HTML version was correct from the time of publication.

