# Supplementary material for: Paracrine interactions between mesenchymal stem cells and macrophages are regulated by 1,25-dihydroxyvitamin D3
Source: Sci Rep. 2017 Nov 3;7:14618. doi: 10.1038/s41598-017-15217-8 (PMC5668416; doi:10.1038/s41598-017-15217-8)
Supplement: Supplementary file 1 — Supplementary Information [file 41598_2017_15217_MOESM1_ESM.pdf]

Paracrine interactions between mesenchymal stem cells and macrophages are regulated by 1,25-dihydroxyvitamin D3

Laura Saldaña[1,2]; Gema Vallés[1,2]; Fátima Bensiamar[1,2]; Francisco José Mancebo[2,1]; Eduardo García-Rey[1]; Nuria Vilaboa[1,2]*

[1]*Hospital Universitario La Paz-IdiPAZ, Paseo de la Castellana 261, 28046 Madrid, Spain*

[2]*CIBER de Bioingeniería, Biomateriales y Nanomedicina (CIBER-BBN), Spain*

Supplementary Table S1. Primer sequences used in PCR.

	Primer Sequence (5'-3')	
Gene	Forward	Reverse
VDR	GCCCACCATAAGACCTACGA	AGATTGGAGAAGCTGGACGA
CBFA1	ATGATGACACTGCCACCTCTGA	GGCTGGATAGTGCATTCGTG
ALPL	GACTAAGAAGCCCTTCACTGCCAT	GACTGCGCCTGGTAGTTGTT
BGLAP	GGCGCTACCTGTATCAATGG	GATAGGCCTCCTGAAAGCCG
OPN	TGAAACGAGTCAGCTGGATG	TGAAATTCATGGCTGTGGAA
OPG	GTGTCTATACTGCAGCCCCGT	GTATTTCGCTCTGGGGTTCCA
RANKL	AGAGCGCAGATGGATCCTAA	TTCCTTTTGCACAGCTCCTT
TNFA	TCCTTCAGACACCCTCAACC	AGGCCCCAGTTTGAATTCTT
IL10	GCCTAACATGCTTCGAGATC	TGATGTCTGGGTCTTGGTTC
VEGFA	GACCCTGGTGGACATCTTCC	CACAGGGATTTTCTTGTCTTGC
IL6	CCCCAGGAGAAGATTCCAAA	CCAGTGATGATTTTCACCAGG
COX2	TGAGCATCTACGGTTTGCTG	TGCTTGTCTGGAACAACTGC
MCP1	CCCCAGTCACCTGCTGTTAT	TGGAATCCTGAACCCACTTC
MIP1A	TGCAACCAGTTCTCTGCATC	TTTCTGGACCCACTCCTCAC
β2M	CCAGCAGAGAATGGAAAGTC	CCAGCAGAGAATGGAAAGTC

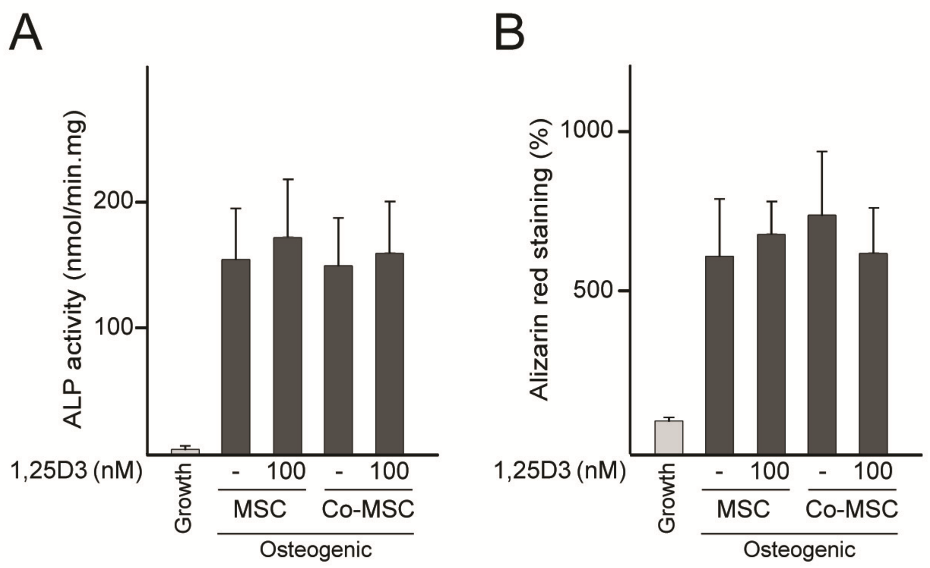

Supplementary Figure S1. Osteogenic differentiation of MSC after co-culturing with dTHP-1. MSC cultured in isolation or co-cultured with dTHP-1 (Co-MSC) were treated with 100 nM 1,25D3 or vehicle (-) for 72 h, and then induced to undergo osteogenic differentiation in the absence of dTHP-1. ALP activity (A) and quantification of alizarin red staining (B) in layers of MSC undergoing osteogenesis for 7 days or 14 days, respectively. Data in B are relative to those measured in cells cultured in growth medium, which were given the arbitrary value of 100.

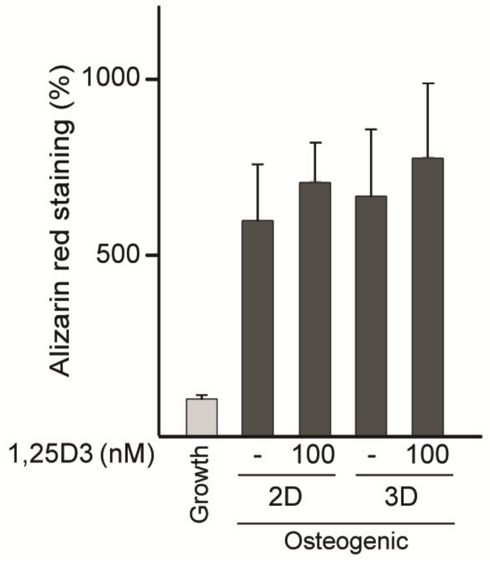

Supplementary Figure S2. Matrix mineralization in MSC after co-culturing with dTHP-1 under 2D or 3D conditions. MSC co-cultured in transwells (2D) or 3D substrates with dTHP-1 were treated with 100 nM 1,25D3 or vehicle (-) for 72 h, and then induced to undergo osteogenic differentiation in the absence of dTHP-1. Quantification of alizarin red staining in layers of MSC undergoing osteogenesis for 14 days. Data are relative to those measured in cells cultured in growth medium, which were given the arbitrary value of 100.

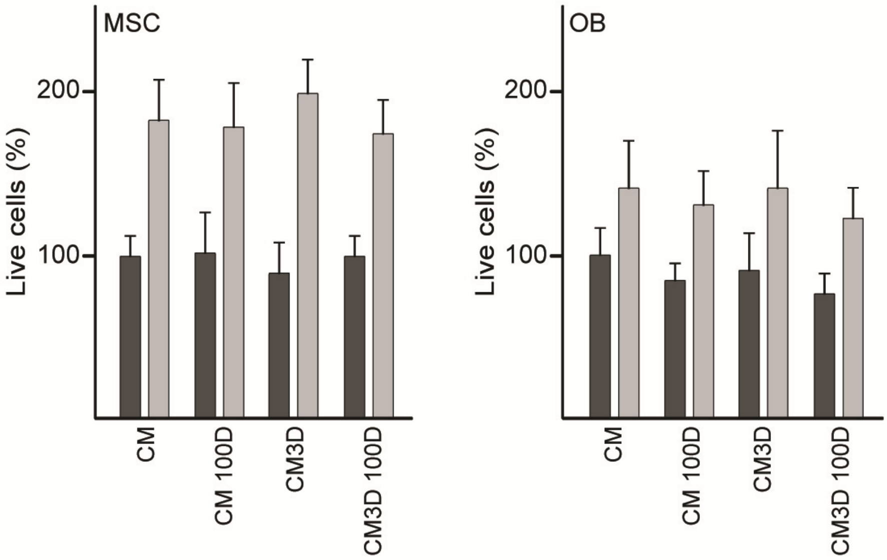

Supplementary Figure S3. Effect of secreted factors from co-cultures on MSC or osteoblast proliferation. MSC or osteoblasts (OB) were cultured for 3 (dark gray) or 7 (light gray) days in conditioned media from 2D co-cultures (CM) or 3D co-cultures (CM3D) treated with 100 nM 1,25D3 (100D) or vehicle. Trypan blue dye exclusion was used to determine the number of live cells. The data are relative to the number of live cells in cultures exposed to CM for 3 days, which were given the arbitrary value of 100.

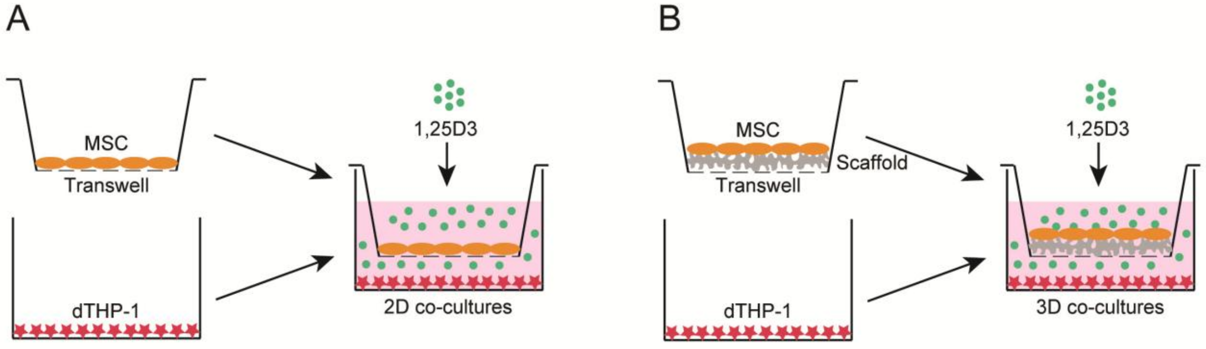

Supplementary Figure S4. Schematic illustration showing the set up of conventional/2D (A) or 3D (B) co-cultures treated with 1,25D3.
